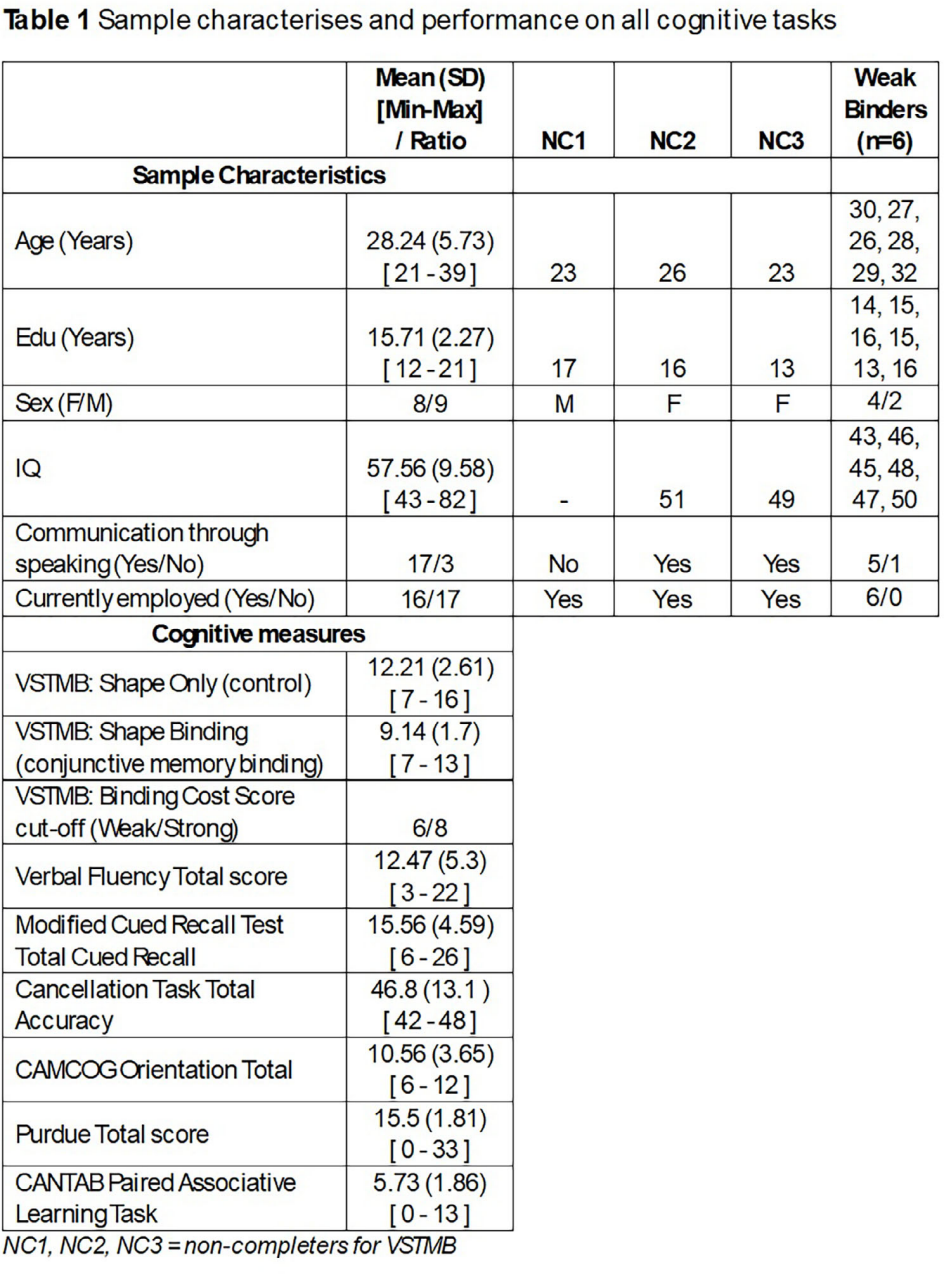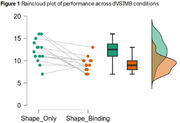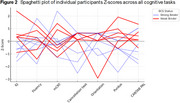# Exploring a preclinical cognitive marker for Alzheimer's disease in adults with Down syndrome: preliminary evidence from a conjunctive memory binding assessment

**DOI:** 10.1002/alz70857_099713

**Published:** 2025-12-24

**Authors:** Tamlyn J Watermeyer, Joe Butler, Samuel O. Danso, John‐Joseph Russell, Mario A Parra‐Rodriguez, The Binding in Neuropsychiatric Disorders (BIND) Collaboration

**Affiliations:** ^1^ Edinburgh Dementia Prevention, Centre for Clinical Brain Sciences, College of Medicine and Veterinary Medicine, University of Edinburgh, Edinburgh, United Kingdom; ^2^ National Institute of Health Applied Research Collaboration North East & Cumbria, Newcastle‐upon‐Tyne, England, United Kingdom; ^3^ University of Northumbria, Newcastle upon tyne, England, United Kingdom; ^4^ School of Psychology, University of Sunderland, Sunderland, United Kingdom; ^5^ National Institute of Health Applied Research Collaboration North East & Cumbria, Newcastle‐Upon‐Tyne, England, United Kingdom; ^6^ School of Computer Science and Engineering, University of Sunderland, Sunderland, England, United Kingdom; ^7^ University of Edinburgh, Edinburgh, United Kingdom; ^8^ Faculty of Health & Life Sciences, Northumbria University, Newcastle‐Upon‐Tyne, England, United Kingdom; ^9^ Universidad Autónoma de Caribe, Barranquilla, Colombia; ^10^ University of Strathclyde, Glasgow, United Kingdom

## Abstract

**Background:**

Adults with Down syndrome (DS) are at a substantially increased risk of Alzheimer's disease (AD), with earlier onset and greater vulnerability (Fortea et al., 2021). However, sensitive neuropsychological tools for detecting early AD in this population are lacking, limiting their inclusion in research trials. Conjunctive memory binding, the ability to integrate and retain multiple features of stimuli, is an early indicator of AD‐related pathology. The visual short‐term memory binding (VSTMB) task assesses this ability, producing a binding‐cost score (BCS) associated with AD neuropathology (Parra et al., 2024). We explore the feasibility, acceptability, and discriminability of a novel digital version of the VSTMB (dVSTMB, Butler, Watermeyer et al., 2024) adapted for DS populations.

**Method:**

Seventeen participants enrolled in the study to complete the dVSTMB and the Horizon 21 Alzheimer's disease (H21AD) battery (Aschenbrenner et al., 2021), recommended by expert consensus for DS‐AD detection. A Bayesian paired‐samples t‐test examined performance differences between the control (Shape‐Only) and experimental (Colour‐Binding) conditions of the dVSTMB. Participants were categorized as “weak” or “strong” binders based on BCS criteria.

**Result:**

Table 1 shows sample characteristics and cognitive performance. dVSTMB completion rate was 82.4%, with non‐completion (*n* = 3) due to difficulty understanding task instructions; no dropouts. Notably, non‐speaking participants (*n* = 3) could complete the dVSTMB, unlike some H21‐AD items. Enjoyment ratings for the dVSTMB were high (M=6.33, SD=0.89, Max=7). Participants performed better in the Shape‐Only condition compared to the Colour‐Binding condition (Figure 1, BF+0=44.28, *pmd* 3.071, 95% CI [2.614; 3.528], *er* 0.002%). BCS status and non‐completion were not influenced by age, education or sex. Individual‐level performance across HS21AD items by BCS status are shown in Figure 2.

**Conclusion:**

These preliminary findings demonstrate initial feasibility and acceptability of dVSTMB for DS populations; however, evidence of difficulty with task instructions for some participants warrants monitoring in larger samples. DS participants’ performances across dVSTMB conditions is in keeping with the expected direction. Some participants met BCS cut‐off criteria indicative of Alzheimer's disease, highlighting its potential for AD screening in DS populations. The pilot study is on‐going, with future work extending to other learning disability populations planned.